# Supplementary material for: Neutron Activation Analysis of Rare Earth Element Extraction from Solution through a Surfactant-Assisted Dispersion of Carbon Nanotubes
Source: Nanomaterials (Basel). 2023 Dec 28;14(1):92. doi: 10.3390/nano14010092 (PMC10780536; doi:10.3390/nano14010092)
Supplement: Supplementary file 1 [file nanomaterials-14-00092-s001.zip › nanomaterials-2750772-supplementary.pdf]

Supporting Information **for**

Neutron Activation Analysis of Rare Earth Element Extraction from Solution through a Surfactant-Assisted Dispersion of Carbon Nanotubes

*Adam Samia**, *Donald Nolting, Joseph Lapka and William Charlton**

School of Nuclear and Radiation Engineering, University of Texas,

Austin, TX 78758-445, USA.

Corresponding Authors

*Email: adam.samia@austin.utexas.edu (Adam Samia)

*Email: wcharlton@austin.utexas.edu (William Charlton)

Submitted to

Nanomaterials

Number of pages: 5

Number of figures: 3

Number of tables: 0

Table of Contents

Supplementary Figures S3

Figure S1. Percent adsorption on the SA-CNTs for each surfactant at varying surfactant concentrations. The plots represent a 20 CNT to 1 Yb mass ratio in (a) GA, (b) Triton X-100, and (c) GO........ S3

Figure S2. SWCNT vs. MWCNT percent adsorption comparison for all three surfactants (15 w/w% GA, 8% Triton X-100, and 1 mg/mL of GO) at a 20 CNT to 1 Yb mass ratio........ S4

Figure S3. CNT stripping percent for Yb with 1.0 M of HCl, 1.0 M of HNO_3, and 0.1 M of DOTA........ S5

Supplementary Figures

Figure S1. Percent adsorption on the SA-CNTs for each surfactant at varying surfactant concentrations. The plots represent a 20 CNT to 1 Yb mass ratio in (a) GA, (b) Triton X-100, and (c) GO.

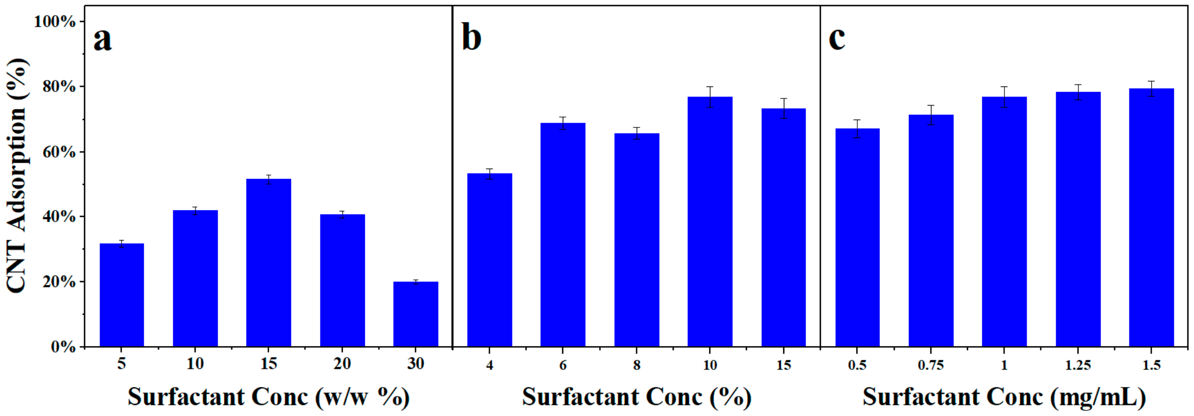

Figure S1. Percent adsorption on the SA-CNTs for each surfactant at varying surfactant concentrations. The plots represent a 20 CNT to 1 Yb mass ratio in (a) GA, (b) Triton X-100, and (c) GO.

Figure S2. SWCNT vs. MWCNT percent adsorption comparison for all three surfactants (15 w/w% GA, 8% Triton X-100, and 1 mg/mL of GO) at a 20 CNT to 1 Yb mass ratio.

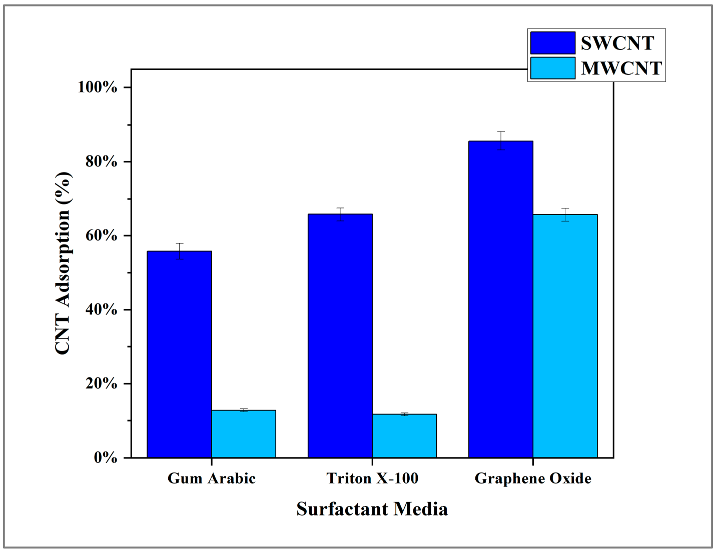

Figure S2. SWCNT vs. MWCNT percent adsorption comparison for all three surfactants (15 w/w% GA, 8% Triton X-100, and 1 mg/mL of GO) at a 20 CNT to 1 Yb mass ratio.

Figure S3. CNT stripping percent for Yb with 1.0 M of HCl, 1.0 M of HNO_3, and 0.1 M of DOTA.

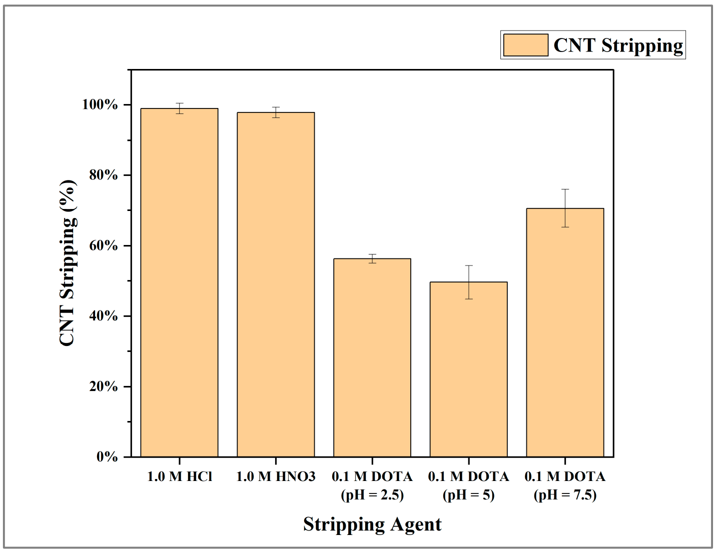

Figure S3. CNT stripping percent for Yb with 1.0 M of HCl, 1.0 M of HNO_3, and 0.1 M of DOTA.
